# Spatial‐EV‐seq: Mapping the spatial organisation of extracellular vesicles in tissues

**DOI:** 10.1002/ctm2.70825

**Published:** 2026-08-31

**Authors:** Xing Xu, Qianxi Wen, Yanling Song, Chaoyong Yang

**Affiliations:** ^1^ Laboratory Technology for Precision Medicine, School of Medical Technology and Engineering Fujian Medical University Fuzhou China; ^2^ The MOE Key Laboratory of Spectrochemical Analysis and Instrumentation, College of Chemistry and Chemical Engineering Xiamen University Xiamen China; ^3^ Hangzhou Institute of Medicine (HIM) Chinese Academy of Sciences Hangzhou China

1

Extracellular vesicles (EVs) are increasingly recognised as promising biomarkers for cancer diagnosis, immunotherapy response prediction, and disease monitoring.^1^, ^2^ Their ability to carry bioactive molecules, including proteins, nucleic acids, and lipids, from parent to recipient cells positions them as critical mediators of intercellular communication. Yet despite this potential, translating EV biology into clinical utility has been hampered by a fundamental limitation: conventional EV analysis relies on tissue dissociation and differential centrifugation, which destroy the spatial architecture of EVs and erase their native distribution, interaction networks, and connections with parent and recipient cells.^3^ The result is a loss of contextual information about where EVs reside, which cells they interact with, and how their location shapes their function.^4^

Spatial transcriptomics has transformed tissue analysis by preserving spatial context while profiling gene expression.^5^, ^6^ But a similar approach for EVs has been conspicuously absent. This gap matters clinically. A growing body of evidence suggests that the location of EVs within tissues is not merely a passive variable but an active determinant of their biological roles. EVs from tumour surfaces carry molecular signatures distinct from those in adjacent tissues, and their spatial heterogeneity reflects disease progression and therapeutic response. To capture this dimension, we developed Spatial‐EV‐seq,^7^ a method for in situ spatial profiling of EVs within their native microenvironmental context.

The technology operates through three integrated steps (Figure 1). First, an antibody‐engineered capture interface immobilises EVs directly from tissue sections via a blotting‐based transfer strategy, preserving their original spatial coordinates with minimal lateral displacement (approximately 5.8 µm) and a recovery rate of 60% to 70%. Second, captured EVs are amplified by rolling circle amplification using EV surface‐binding aptamers, enabling fluorescence imaging of individual EVs at approximately 1 µm resolution. Third, by integrating these spatial EV maps with spatial transcriptomics^8^ on adjacent sections, the method resolves location‐specific EV‐cell communication networks, linking EV position, molecular identity, and neighbouring cellular states in a single analytical framework.

**FIGURE 1:**
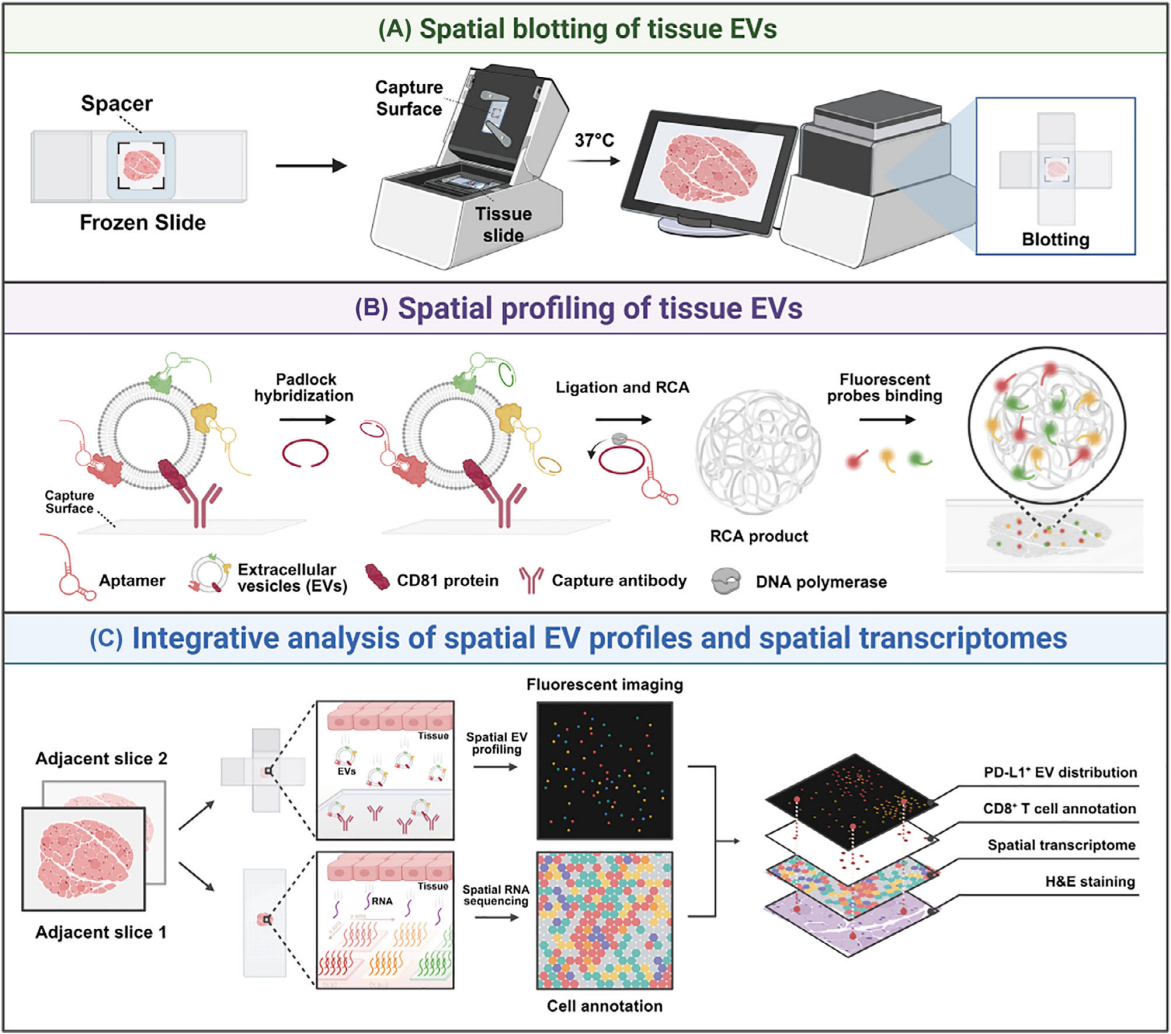
Schematic overview of the Spatial‐EV‐seq workflow. (**A**) EVs are transferred from tissue sections to an antibody‐coated capture surface via a blotting‐based strategy. (**B**) Captured EVs are amplified by rolling circle amplification using EV surface‐binding aptamers, enabling fluorescence imaging of individual EVs. (**C**) Integration of spatial EV maps with spatial transcriptomics on adjacent sections resolves location‐specific EV‐cell communication networks, linking EV position, molecular identity and neighbouring cellular states in a single framework.

Applying Spatial‐EV‐seq to an anti‐PD1‐treated breast cancer mouse model, we uncovered a spatially orchestrated immunosuppressive axis. PDL1^+^ EV‐enriched zones correlated with CD8^+^ T cell exhaustion, establishing immune‐privileged niches, whereas PDL1^+^ EV‐depleted regions preserved immunocompetence and therapeutic sensitivity. Notably, regions with high PDL1^+^ EV abundance but low CD274 gene expression indicated that PDL1^+^ EVs can migrate from parent cells to distal sites, functioning independently of local cellular PDL1 expression. This spatial dissociation underscores a key clinical insight: inferring EV function from cellular gene expression alone is insufficient. Direct spatial visualisation of EVs provides information that transcriptomics alone cannot capture.

The clinical implications are substantial. First, Spatial‐EV‐seq enables spatially resolved biomarker discovery. By mapping EV subpopulations directly in tissue biopsies, the method can identify location‐specific EV signatures associated with immunotherapy resistance or sensitivity. Second, it provides a framework for understanding how EV spatial organisation contributes to tumour heterogeneity and immune evasion, potentially revealing new therapeutic targets. Third, the platform's adaptable capture interface allows customisation for specific EV subtypes or broader profiling with antibody cocktails, making it versatile for diverse clinical questions, from predicting treatment response to monitoring disease progression.

Several limitations merit consideration. Current implementation relies on adjacent tissue sections for EV and transcriptomic analysis, which may introduce registration errors and limit precise EV‐transcriptome correspondence. The fluorescence‐based readout also constrains multiplexing capacity, typically allowing only several markers to be detected simultaneously. Future developments, particularly integrating EV detection and transcriptomics on the same tissue section, and converting EV signals into sequencing‐compatible barcodes for high‐throughput decoding, could overcome these barriers and enable comprehensive spatial mapping of dozens to hundreds of EV surface proteins and cargo molecules in a single experiment. The method's current spatial resolution, approximately 1 µm for EV imaging and 50 µm for transcriptomics, is sufficient for tissue‐region‐level analysis, but further refinement may enable single‐cell‐level integration as spatial technologies evolve.

Looking ahead, Spatial‐EV‐seq opens several translational avenues. In precision oncology, it could guide biopsy‐based patient stratification by revealing the spatial organisation of immunosuppressive EV niches. In drug development, it offers a tool to assess how therapeutic agents alter EV spatial distribution and function within tumours. In basic research, it provides a platform to systematically interrogate how EV spatial heterogeneity contributes to disease mechanisms across cancer types and tissue contexts.

By moving EV analysis from ‘bulk homogenate’ to ‘in situ spatial resolution’, Spatial‐EV‐seq establishes a new paradigm for understanding how the spatial organisation of EVs contributes to tissue homeostasis, disease progression, and therapeutic responses, bringing the field one step closer to leveraging EV biology for precision medicine.

## CONFLICT OF INTEREST STATEMENT

The authors declare no conflicts of interest.
